# The Impact of Al_2_O_3_ Particles from Grit-Blasted Ti6Al7Nb (Alloy) Implant Surfaces on Biocompatibility, Aseptic Loosening, and Infection

**DOI:** 10.3390/ma16216867

**Published:** 2023-10-26

**Authors:** Boštjan Kocjančič, Klemen Avsec, Barbara Šetina Batič, Darja Feizpour, Matjaž Godec, Veronika Kralj-Iglič, Rok Podlipec, Andrej Cör, Mojca Debeljak, John T. Grant, Monika Jenko, Drago Dolinar

**Affiliations:** 1 Department for Orthopaedic Surgery, UMC Ljubljana, Zaloška 9, 1000 Ljubljana, Slovenia; kocjancicb@gmail.com (B.K.); kavsec@gmail.com (K.A.); dolinardrago@gmail.com (D.D.); 2 Chair of Orthopedics, Faculty of Medicine, University of Ljubljana, Vrazov trg 2, 1000 Ljubljana, Slovenia; 3 Institute of Metals and Technology, Lepi pot 11, 1000 Ljubljana, Slovenia; barbara.setina@imt.si (B.Š.B.); darja.feizpour@imt.si (D.F.); matjaz.godec@imt.si (M.G.); 4 University of Ljubljana, Faculty of Health Sciences, Laboratory of Clinical Biophysics, 1000 Ljubljana, Slovenia; veronika.kralj-iglic@zf.uni-lj.si; 5 Laboratory for Biophysics, Jozef Stefan Institute, Jamova 39, 1000 Ljubljana, Slovenia; rpodlpec@ijs.si; 6 Ion Beam Center, Helmholtz-Zentrum Dresden-Rossendorf e.V., 01328 Dresden, Germany; 7 Orthopaedic Hospital Valdoltra, Jadranska cesta 31, 6280 Ankaran, Slovenia; andrej.cor@ob-valdoltra.si; 8 University Rehabilitation Institute Republic of Slovenia Soča, Linhartova 51, 1000 Ljubljana, Slovenia; mojca.debeljak@ir-rs.si; 9 Research Institute, University of Dayton, Dayton, OH 45469, USA; john.grant@surfaceanalysis.org; 10 MD-RI Institute for Materials Research in Medicine, Bohoričeva 5a, 1000 Ljubljana, Slovenia

**Keywords:** Ti6Al7Nb implant alloy cementless hip endoprostheses, roughness, Al_2_O_3_ grit blasting, surface and subsurface implant contamination, cytotoxicity, aseptic loosening, infection, osseointegration

## Abstract

For the improvement of surface roughness, titanium joint arthroplasty (TJA) components are grit-blasted with Al_2_O_3_ (corundum) particles during manufacturing. There is an acute concern, particularly with uncemented implants, about polymeric, metallic, and corundum debris generation and accumulation in TJA, and its association with osteolysis and implant loosening. The surface morphology, chemistry, phase analysis, and surface chemistry of retrieved and new Al_2_O_3_ grit-blasted titanium alloy were determined with scanning electron microscopy (SEM), X-ray energy-dispersive spectroscopy (EDS), transmission electron microscopy (TEM), X-ray photoelectron spectroscopy (XPS) and confocal laser fluorescence microscopy, respectively. Peri-prosthetic soft tissue was studied with histopathology. Blasted retrieved and new stems were exposed to human mesenchymal stromal stem cells (BMSCs) for 7 days to test biocompatibility and cytotoxicity. We found metallic particles in the peri-prosthetic soft tissue. Ti6Al7Nb with the residual Al_2_O_3_ particles exhibited a low cytotoxic effect while polished titanium and ceramic disks exhibited no cytotoxic effect. None of the tested materials caused cell death or even a zone of inhibition. Our results indicate a possible biological effect of the blasting debris; however, we found no significant toxicity with these materials. Further studies on the optimal size and properties of the blasting particles are indicated for minimizing their adverse biological effects.

## 1. Introduction

Total joint arthroplasty (TJA) replacement surgeries have been very successful for decades [1]. The most commonly used implant materials for hip endoprostheses in orthopedics are Ti6Al7Nb and Ti6Al4V alloys (more information is given in [2]). The Zweymüller (ZM) uncemented hip endoprosthesis made of forged titanium alloy Ti6Al7Nb, with the double-taper SL-PLUS^®^ femoral stem with a rectangular cross-section and grit-blasted 4–8 μm surface roughness to enhance bone in-growth, has been used for the last 30 years with an unchanged design [3]. Roškar et al. reported on a 27 years follow-up on the clinical outcome for 2129 titanium swindler SL-PLUS^®^stem femoral stems [3]; the presented data corroborate the excellent results for the ZM TJA obtained from arthroplasty registries [3].

However, somewhat less than 10% of THJ fail in the first 10–20 years, with aseptic loosening and peri-prosthetic joint infection as the main causes [3,4,5]. Aseptic loosening can be induced due to micromotion of the implant in the bone during loading and due to corundum wear particles from grit-blasted surfaces which may cause inflammation and bone resorption, and consequently the formation of a poor functional interface between the implant and the patient’s bone [6]. This indicates the importance of a better understanding of the mechanisms taking place at the interface between cells and foreign materials as well as the findings obtained in bone tissue engineering [7,8].

The population is aging, and life expectancy is increasing, so besides premature failures, many patients are outliving their implants. Both factors predict a large increase in implant failure in the future and an enormous increase in health costs. It is necessary to extend the longevity of implants, to reduce the number of revisions, and to reduce the health costs.

Nowadays, there are approximately 60 different types of single stem cementless prostheses on the market [4], and understanding the behaviour of the prosthesis types in given clinical conditions is very important. Merola and Affatato reported that monitoring the use of prosthetic devices and ensuring the traceability of the patients in the case of adverse events is very difficult [4]. Infections of the implant surface occur when bacteria attach to the implant surfaces, and form a biofilm hindering their elimination [9,10]. Balaban et al. [10] reported that particulate bacteria species are responsible for most pathogens. *Staphylococcus aureus* and *epidermidis* cause almost 70% of orthopedic implant infections [10,11,12].

The aim of this investigation was a detailed study of the physico-chemical phenomena on the surfaces and subsurfaces of the Ti6Al7Nb alloy cementless hip endoprostheses. Our goal was to distinguish the causes of premature failure of aseptic loosening and periprosthetic infection and to determine the role of particulate corundum debris in these issues. Quinn et al. [13] reported that the main causes for premature failed implants are (i) infection, (ii) mechanical failure, (iii) mismatch in elastic modulus, (iv) excessive wear, (v) surgical failure, and (vi) inflammatory response. Quinn et al. [13] also reported on factors affecting the biocompatibility: (i) topography, (ii) elastic modulus, (iii) specific strength, (iv) cytotoxicity, (v) surface charge, (vi) wettability, (vii) surface free energy, and (viii) corrosion resistance.

Here, we focused on premature failed Total Hip Arthroplasty (THA) due to aseptic loosening and periprosthetic infection. We investigated the grit-blasted surfaces of cementless ZM-type stems produced from Ti6Al7Nb alloy for which the corrosion resistance of the corundum grit-blasted and polished surface was previously investigated in electrochemical experiments, carried out in a simulated physiological Hank’s solution [2,14,15] and the surface wettability was determined with contact angle and roughness [16].

The tissue adjacent to the retrieved prosthesis was taken for histopathological analysis. We have focused on the cytotoxicity and cell response (according to ISO 10933-5, https://www.iso.org/standard/36406.html/, accessed on 20 October 2023) of cementless stems with polished surfaces and rough surfaces after grit blasting and on the materials used during stem manufacturing (e.g., forged Ti6Al7Nb and corundum granulate WFA46). The cleaning and sterilization processes, according to the literature data and our experiences, do not affect the removal of the particle debris to be studied [17,18].

One of the major unresolved problems in joint replacement is the adverse biological reaction to implant materials and wear debris that can activate the immune response and thus cause severe inflammation [15].

## 2. Materials and Methods

All procedures performed in studies involving human participants were in accordance with the ethical standards of the institutional and/or national research committee (the National Medical Ethics Committee of the Republic of Slovenia, with the revised Helsinki Declaration of 2013) and comparable ethical standards. The permission (case No. 0120-457/2017/3) was obtained on 22 August 2017.

The investigation included 32 stems of cementless hip endoprostheses that prematurely failed due to (i) aseptic loosening (10 implants, Group A), (ii) infection (10 implants, Group I), and (iii) low-grade infection (10 implants, Group L) and 2 new stems after their expiry date. The interval between the primary hip replacement and the revision surgery ranged from 36 months to 259 months for aseptic loosening, 3 months to 40 months for infection, and 12 months to 198 months for low-grade infection. The retrieved stems were the ZM type from an unknown manufacturer, while the new ones were from Zimmer and Smith & Nephew—a worldwide known manufacturer of Ti6Al7Nb stems for cementless hip endoprostheses. An image of the retrieved components and the available data on them are presented in Appendix A.

The retrieved implants were subjected to sonication and microbiological analyses in a Ringer’s solution followed by cleaning and sterilization. All the retrieved stems were cleaned according to standard procedures at the Ljubljana University Medical Centre, which consisted of immersion in a 2% micro soap solution, followed by acetone, isopropanol (xN), 95% ethanol (xN), and deionized water (xN); xN is the number of repeated processes. Sterilization was performed with autoclaving according to a standard protocol at 120 °C and a pressure of 1.25 bar for 20 min. Afterwards, sterilized stems were kept in sterile bags in a dry place for further investigations. New femoral components were cleaned and sterilized at the manufacturer’s site and special bags were opened on site just before surface chemistry and microstructure investigation. The cleaning and sterilization processes, according to the literature data and our experiences, do not affect the removal of the particle debris to be studied [16,19].

**Figure 1:**
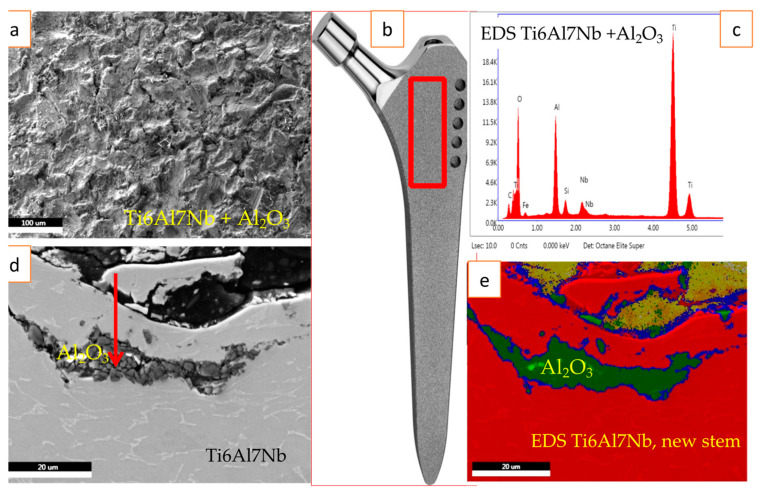
(**a**) SE image of rough surface (S_a_ 6 μm); (**b**) cementless femoral component of a new ZM hip endoprosthesis stem; the retrieved stems after being cleaned and sterilized appear the same; the red box indicates the region investigated; (**c**) EDS spectrum of a representative sample indicating that the dark grey region in SE image (**d**) and green in (**e**) is corundum Al_2_O_3_; (**d**) SE image of a cross-section of new Ti6Al7Nb stem indicating residual Al_2_O_3_ surface contamination (dark grey) in the subsurface up to 20μm from an Al_2_O_3_ grit blasting; (**e**) EDS mapping showing the Ti6Al7Nb matrix (red) and Al_2_O_3_ contamination (green). Different colors correspond to different materials.

From the revision surgeries, we selected three retrieved Ti6Al7Nb stems of the cementless ZM type of hip endoprostheses from each group, and for the comparison, we investigated new stems from different manufacturers (after their expiry date) as described in previous papers [16,19,20]. Representative results on the composition of the stem surfaces from each group are shown in Figure 1.

We performed surface chemistry and surface analysis with scanning electron microscopy (SEM), X-ray energy-dispersive spectroscopy (EDS), transmission electron microscopy (TEM), and X-ray Photoelectron Spectroscopy (XPS).

Wear particles and lymphocytes in tissue around failed joint prostheses were studied with histopathology [16,21]. The patient’s demographic information, including sex, age at index THA, body-mass index, survivorship of implantation, and reason for revision, was collected from medical records.

For cytotoxicity investigation, we retrieved the femoral component of the cementless hip endoprosthesis (Ti6Al7Nb, ZM stem) that underwent aseptic loosening, sterilized it, acquired material from the surface, processed it with corundum, and exposed it to cells in culture. For control, the same procedure was applied to material from a new polished stem. Also, corundum disks and corundum micro-particles were exposed and tested for cytotoxicity.

For the morphology, microstructure, and surface chemistry, the samples were analyzed using a field-emission scanning electron microscope (ZEISS crossbeam 550 FIB-SEM Carl Zeiss AG, Oberkochen, Germany). The instrument is equipped with secondary-electron (SE), backscattered-electron (BE) imaging modes for analyses of the morphology of the samples and energy-dispersive x-ray spectroscopy (EDS) (EDAX, Octane Elite, Draper, Cambridge, MA, USA) for the surface chemistry (3 μM). For the SE and BE imaging an acceleration of 15 kV at a current of approximately 0.5 nA was used at a vacuum below 10^−6^ mbar.

The X-ray photoelectron spectroscopy (XPS) analyses were carried out on the PHI-TFA XPS spectrometer from Physical Electronics Inc., Chanhassen, MN, USA. Samples were polished before the analyses and exposed to air for one day. The native oxide layer was analyzed for thickness and composition on two Ti6Al7Nb samples. The analyzed area was 0.4 mm in diameter and the analyzed depth was about 3–5 nm. This high surface sensitivity is a general characteristic of the XPS method. Sample surfaces were excited with X-ray radiation from a monochromatic Al source at a photon energy of 1486.6 eV. Quantification of the surface composition was performed from the XPS peak intensities, considering the relative sensitivity factors provided by the instrument manufacturer [22]. We estimate that the relative error of the calculated concentrations is about 20% of the reported values. To analyze the in-depth distribution of elements in the subsurface region up to 25 nm, XPS depth profiling was performed in combination with argon ion sputtering. Argon ions with 3 keV of energy were used. The rate of the ion sputtering was estimated to be 1.0 nm/min, calibrated on a Ni/Cr multilayer structure of known thickness.

Transmission electron microscopy (TEM) was performed with a JEOL JEM-2200FS HR (JEOL, Tokyo, Japan) electronic microscope operating at 200 kV. The collected powder samples with a volume of about 0.25 mL were dispersed in 1 mL ethanol. A drop of this suspension was put on a copper TEM grid with an amorphous carbon film. The grids were then dried before they were used for TEM investigations. Phase composition and crystal structure of the milled powder sample were determined with XRD analysis with an X-ray diffractometer D4 ENDEAVOR (Bruker AXS GmbH, Karlsruhe, Germany) using CuKα radiation (2θ: 5–80°, step: 0.040°, step time: 6 s).

For cytotoxicity investigations, all materials were sterile and packaged separately. The granulation of Al_2_O_3_ corundum micro-particles was 300–400 μm (WFA 40). The material of the stems was polished and grit-blasted Ti6Al7Nb. The materials were exposed to a human primary mesenchymal stromal (stem) cell culture derived from human bone marrow (BMSCs) in a direct contact system (ISO 10993-5:2009, https://www.iso.org/standard/36406.html/, accessed on 20 October 2023). The materials investigated were fixed to the bottom of the cell culture vessel and BMSCs were seeded evenly throughout the vessel (including the biomaterial surface). The samples were incubated at 37 °C and 5% CO_2_ for 7 days. The cells in the vicinity of the material were monitored for their morphology, and after 7 days the materials were stained with tetrazolium dye MTT (3-(4,5-dimethylthiazol-2-yl)-2,5-diphenyltetrazolium bromide) and inspected for the presence of live cells. The negative (non-cytotoxic) and positive (cytotoxic) controls were in-house materials, validated with ISO 10993 proposed controls. The effect of Al_2_O_3_ corundum on the morphology of BMSC cells was also studied by fixing BMSC cells overnight in a fixative gold sputtering and inspecting the samples with a dual beam scanning electron microscope (SEM) Zeiss Gemini 550 (Oberkochen, Germany).

The *in vitro* cell growth and proliferation study of BMSC primary stem cells derived from the human bone marrow was performed on the same base material with the same thermomechanical history but with different surface finishing. Confocal 2D/3D laser scanning fluorescence microscopy was used to observe the growth of live cells. After gamma sterilization and cell seeding onto materials, each sample was put into an individual measuring chamber, adding a 500 μL volume suspension with 10^5^ cells/mL concentration, samples were incubated for 7 days prior to fluorescence microscopy measurements. Cells were stained with Cell Mask Orange and SiR Actin (1 μM concentrations), both from Invitrogen (Carlsbad, CA, USA), 1 h prior imaging for visualization of cell membranes and actin cytoskeleton using 561 nm and 640 nm lasers and 2 channel detection.

For the histological study, the tissue samples obtained during the revision arthroplasty were fixed in formalin for 24 h and embedded into paraffin. From each paraffin block, 5 μM thick paraffin sections were cut and stained with hematoxylins and eosin. Tissue slides were observed using a microscope with light polarization (Eclipse 80i, Nikon, Tokyo, Japan). Representative images from histological slides were captured with a camera (DS-U3, Nikon, Tokyo, Japan) and analyzed.

## 3. Results and Discussion

### 3.1. Surface and Subsurface Contamination of Ti6Al7Nb Alloy via Al_2_O_3_ Grit Blasting

We studied the surface and subsurface of new and retrieved cementless ZM-type femoral stems of the hip endo-prostheses from different producers that prematurely failed due to aseptic loosening, periprosthetic infection, and latent infection. Previously, by using SEM (JEOL JSM 6500F field-emission scanning electron microscope (Peabody, MA, USA)) we were the first to discover that the subsurface corundum contamination of all the investigated new and retrieved Ti6Al7Nb alloys extended down to a depth of 20 μM [2,16,23]. These cracks present favorable sites for the adhesion of bacteria thereby causing implant infection and loosening. It is therefore indicated that besides *aseptic loosening*, the corundum particles can participate in *implant infection* and intergranular or crevice corrosion.

The rough surface was obtained with an Al_2_O_3_ grit blasting procedure performed on a new stem made of Ti6Al7Nb alloy; the cross-section of the same surface showed subsurface contamination with the embedded Al_2_O_3_ debris particles, shown in Figure 1. We found subsurface Al_2_O_3_ contamination on the samples that prematurely failed due to aseptic loosening and periprosthetic infection.

**Figure 2:**
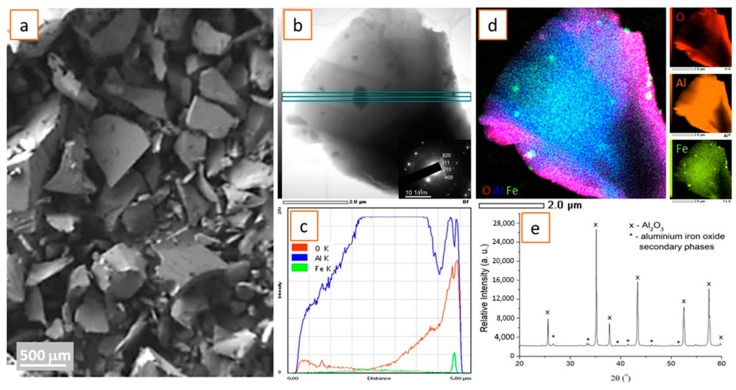
(**a**) SE image of commercial white fused alumina WFA 40 of irregular granulation 300–400 μM for surface roughening of implants; (**b**) Bright-field (BF) TEM image of highly purified white corundum particle with marked TEM profile analysis and inset of electron diffraction; (**c**) TEM profile analysis shows Al, O, and Fe traces in the corundum particle; (**d**) TEM mapping analysis with Al, O, and Fe elements; and (**e**) XRD spectrum of highly purified white corundum milled powder.

We analyzed the commercially available WFA40 (White Fused Alumina) used for grit blasting processes in medicine, using SEM, as shown in Figure 2a, TEM (Figure 2b–d), and XRD (Figure 2e). The SEM image shows powder particles or irregular granulation at 300–400 μm. Using TEM and selected area electron diffraction (SAED) on smaller electron-transparent samples, the 210, 311, 400, and 620 planes were determined in the chosen particle (Figure 2b), which, in addition to profile (Figure 2c) and elemental (Figure 2d) analysis using STEM/EDS, confirmed the presence of aluminum (Al) and oxygen (O) in the sample, with traces of iron (Fe) (likely remnants from bauxite ore [24]). According to the JCPDS-ICDD powder diffraction card (JCPDS-ICDD number 00-010-0173 X’Pert High Score Plus Version 2.2b.2006), the main phase detected with the X-ray detection limit was Al_2_O_3_, known as alpha alumina (Figure 2e). A small number of secondary phases based on aluminum iron oxide solid solutions (such as AlFeO_3_ (JCPDS-ICDD no. 00-030-0024) and Al_3_Fe_5_O_12_ (JCPDS-ICDD no. 00-049-1657)) were also observed, which could be attributed to the impurities in the initial aluminum oxide precursor. Some weak unmarked XRD peaks came from W radiation and not from the main and secondary phases, since during XRD analysis W vaporizes on the Cu target and this causes weak peaks at 2θ = 33.6°, 41.5°, 50.1°, and 54.8°.

**Figure 3:**
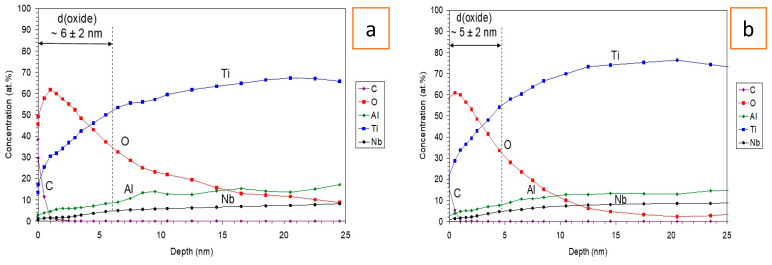
(**a**) XPS depth profile of a new Ti6Al7Nb alloy covered with native oxide after polishing. The thickness of the oxide was estimated to be 6 ± 2 nm. (**b**) XPS depth profile of a retrieved Ti6Al7Nb alloy covered with a native oxide after polishing. The thickness of the oxide was estimated to be 5 ± 2 nm.

The surface analysis of the retrieved and new stems was performed with XPS. Figure 3 shows XPS depth profiles of native oxide film which forms on the fresh surface, after grinding and polishing and exposure to the air for 24 h, of new and retrieved stems. The oxide layer on new and retrieved samples consisted of Ti-oxide, Al-oxide, and Nb-oxide. This was evidenced by the Ti 2p_3/2_ peak at 458.6 eV, characteristic of Ti (4+) in a TiO_2_-like environment; the Nb 3d_5/2_ peak at 207.0 eV, characteristic of the Nb (5+) oxidation state; and the Al 2p peak at 74.0 eV, characteristic for the Al (3+) oxidation state.

### 3.2. Light Microscopy Biocompatibility–Cytotoxicity Studies

One of the major unresolved problems in joint replacement is the adverse biological reaction to implant materials that can activate the immune response and thus cause severe inflammation [15,25].

#### 3.2.1. Characterization of Cytotoxicity

**Figure 4:**
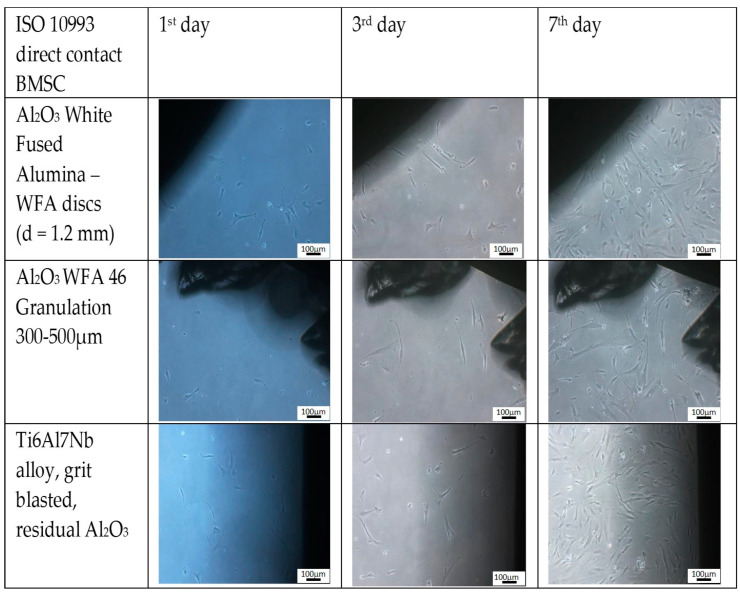
The results of daily monitoring of cytotoxicity testing. The photos represent the cells, grown in immediate vicinity of the materials. The Al_2_O_3_ disc shows no sign of cytotoxicity. Corundum small particles, except for the + control, exhibit the most obvious cytotoxic effect. The Ti6Al7Nb alloy with a modified surface via grit blasting and with residual debris particles on the surface and subsurface exhibited a small level of cytotoxicity but only on the surface where a corundum cover was present. When those samples were polished, there was no cytotoxic effect (not shown).

We studied the interface between the biomaterial and biosystem osseointegration. Growth of cells on different surfaces is shown in Figure 4. 

**Figure 5:**
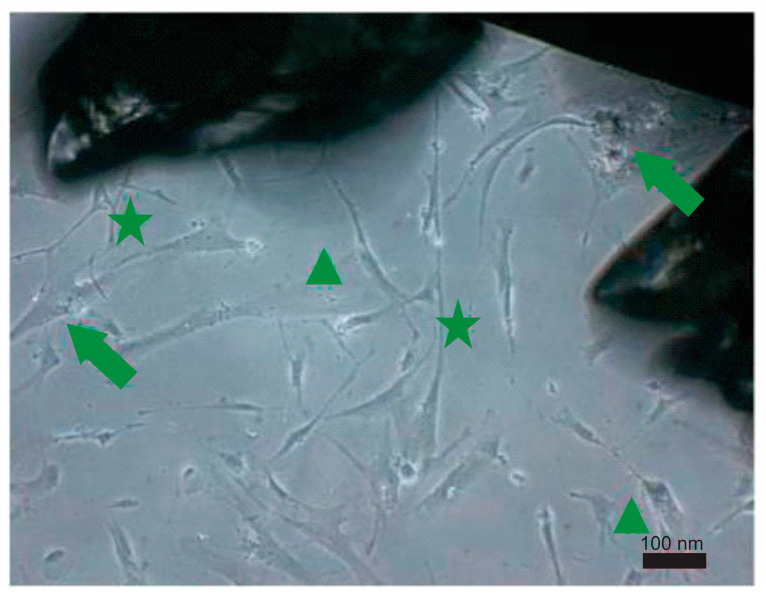
Signs of stress in the vicinity of corundum small particles: inclusion bodies (arrows), overspread cells (stars), and overspread cells (triangles) on a Ti6Al7Nb alloy with a modified surface via grit blasting and with residual debris particles on the surface and subsurface.

The Al_2_O_3_ disc showed no sign of cytotoxicity from corundum small particles while the positive control exhibited an obvious cytotoxic effect. The Ti6Al7Nb alloy with a modified surface via grit blasting and with residual debris particles on the surface and subsurface showed a small level of cytotoxicity, but only on the surface where the corundum cover was present (Figure 5). When those samples were polished, there was no cytotoxic effect.

No cytotoxicity was observed in a similar study on human umbilical vein endothelial cells (ref. [26]) which is in line with the results presented in this work.

#### 3.2.2. Effect of Al_2_O_3_ Corundum on Morphology of Human Bone Marrow-Derived Mesenchymal Stromal Cells (BMSCs)

**Figure 6:**
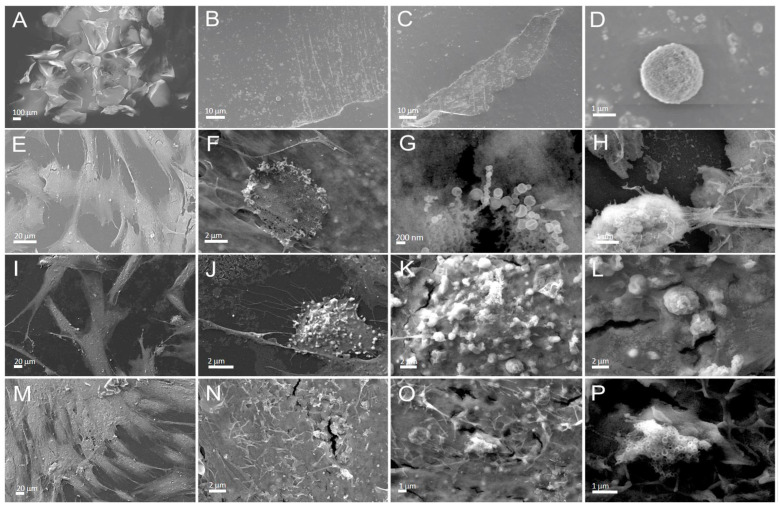
The effect of residual Al_2_O_3_ particles on the morphology of BMSC cells. (**A**–**D**) Corundum particles on the glass surface, (**E**–**H**) cells growing one week on corundum ceramic disc, (**I**–**L**): cells growing on glass with added toxic compound (positive control), and (**M**–**P**) cells growing on glass (negative control).

In Figure 6, the first row (A–D) shows particulate matter distributed over a thin film. The majority of particles are within micrometer size; however, singular larger particles could be found (Panel D). The second row (E–H) shows cells grown on an Al_2_O_3_ ceramic disk. The cells proliferated (Panel E); however, we found many decaying cells, either in the form of vesicles (Panels F and G) composed in a ring-like form (Panel F) or rounded and deformed (possibly apoptosis or necrosis). The third row shows cells growing on glass but with the addition of toxic compounds. Some of the cells proliferated to about the same extent as the ones growing on the corundum disc (Panels E and I). Many rounded decaying cells were found (Panels J–L) (probably apoptosis). The fourth row (Panels M–P) shows negative controls growing on the glass. Cells proliferated well (Panel M), forming more than one layer. Nanotubular structures characteristic of proliferating cells were abundant (Panels N and O). Interestingly, some colloid-like material was found in the sample (Panel P). We found that titanium after corundum grit blasting and the corundum microparticles exhibited a low cytotoxic effect, while polished titanium and ceramic disks exhibited no cytotoxic effect. None of the tested materials in our setup caused cell death or even a zone of inhibition, which indicates that none of the materials are significantly cytotoxic. In a study on human umbilical vein endothelial cells, no cytotoxicity was observed [26] which is in line with the presented results. The present cytotoxicity investigation with primary BMSCs shows that retained corundum causes cell stress which might affect osseointegration.

Preliminary *in vivo* studies of growth in living BMSC primary stem cells derived from human bone marrow BMSC planted onto the same base material Ti6Al7Nb stem alloy with the same thermomechanical history and different surface finishing, such as (i) a Ti6Al7Nb polished surface (mirror-like), (ii) a Ti6Al7Nb ground surface, and (iii) a Ti6Al7Nb Al_2_O_3_ grit-blasted surface, with retained Al_2_O_3_ debris (up to 20%) and a roughness of 4–6 μM, were performed. Confocal 2D and 3D laser fluorescence microscopy was used to observe the growth of live cells. We measured cell growth, attachment, and morphology after several days. A 2D and 3D multichannel confocal fluorescence microscope was used to study living cells and the effect of different materials on growth.

**Figure 7:**
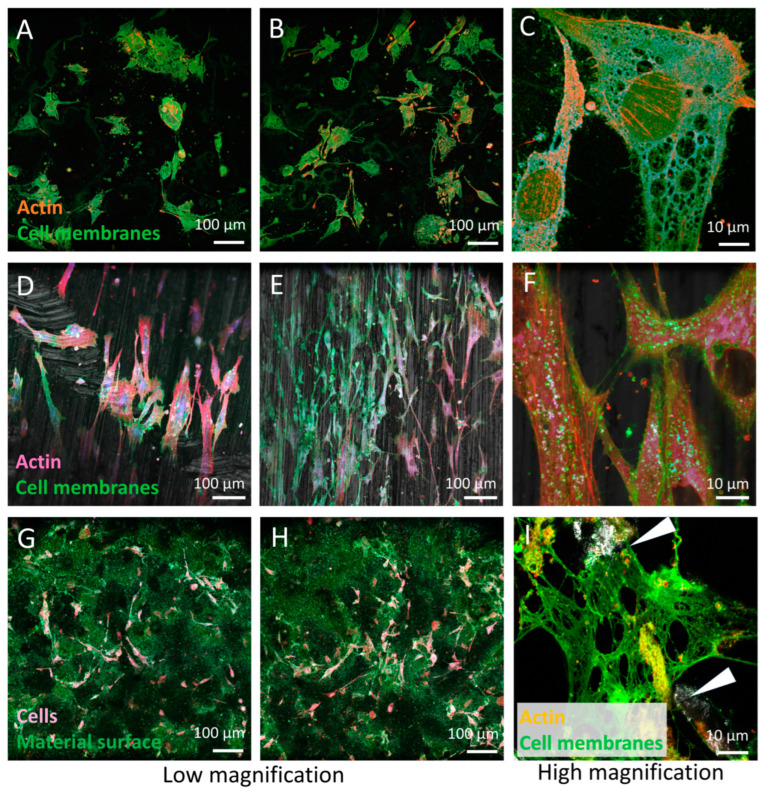
Confocal fluorescence images of live BSMCs grown on Ti6Al7Nb alloys with different surface finishing. (**A**–**C**) BMSCs on a polished (mirror-like) surface with non-oriented growth; (**D**–**F**) BMSCs on a ground surface with oriented growth; (**G**–**I**) BMSCs on a rough, Al_2_O_3_ contaminated surface with non-oriented poor growth, mostly only partially attached to the surface via anchoring points (see the arrows).

**Figure 8:**
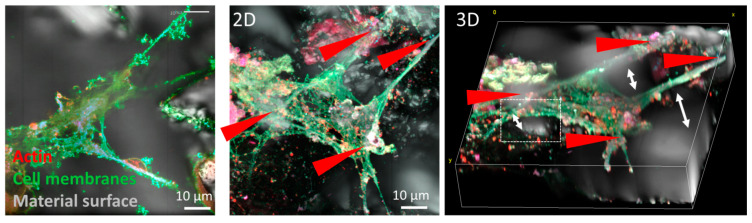
Confocal 2D/3D fluorescence microscopy of locally adhered live BSMCs on the rough Ti6Al7Nb surface with remaining Al_2_O_3_ particles embedded. The material surface (gray color) is observed through the backscatter detection of the laser source. In this example, cells are adhered to the surface through four anchoring sites (red arrows), where the gaps between the surface and the cell are denoted with a white arrow and dashed rectangle. The gaps could be a possible place for bacteria adherence and consequently premature failure due to periprosthetic infection.

Figure 7 and Figure 8 show that BMSC cell growth is different on materials with different surface finishing. There is no preferred orientation of cells on the polished (mirror-like) surface; while on the ground surface, the cells are oriented in the grinding direction.

The effect of the surface on the growth of BMSCs is outlined in Table 1. While the cells adhered and grew nicely on the polished and ground surfaces and resisted infection, they were less numerous and prone to infection when growing on the surface with residual Al_2_O_3_ debris.

### 3.3. Histopathology Studies

**Figure 9:**
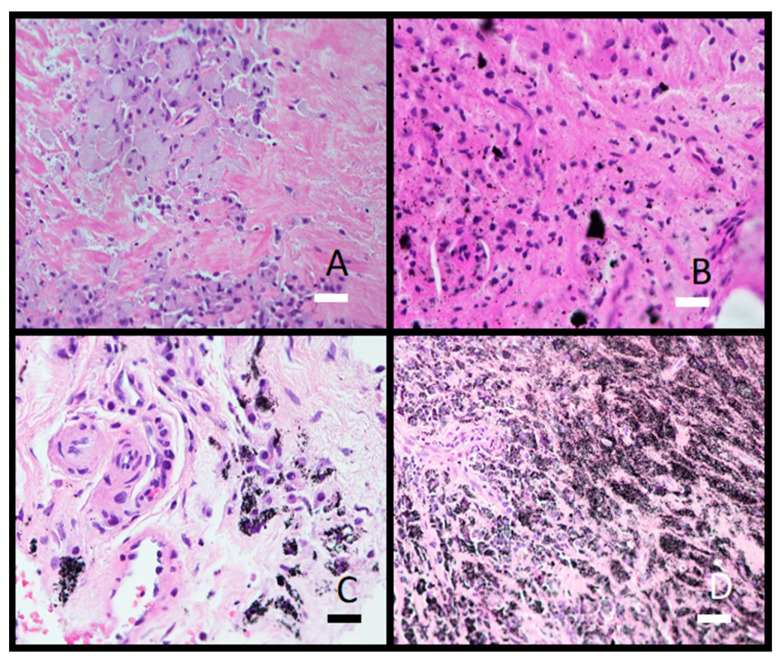
Photomicrographs of granulomatous tissue from a periprosthetic membrane filled with macrophages containing metal wear particles. (**A**) Gray-blue macrophage cytoplasm (obj. mag. 20×); (**B**) Black, needle-shaped to polygonal, sharp-edged metal microparticles different in size (obj. mag. 20×); (**C**) Cytoplasm filled with black particles (obj. mag. 40×); (**D**) Severe metallosis with metal particles in macrophages and extracellular space (obj. mag. 20×). Black bar = 50 μm and white bars = 100 μm.

**Figure 10:**
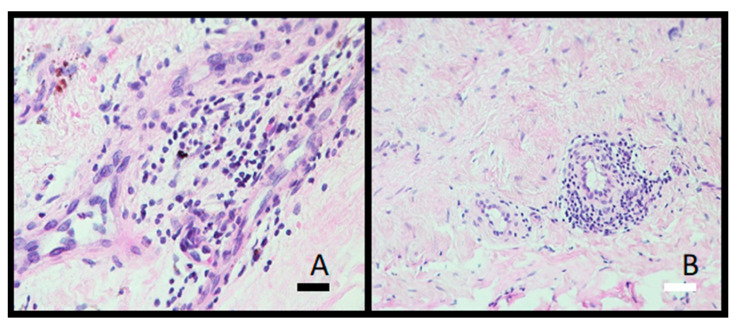
Photomicrograph illustrations of lymphocytic infiltrate in periprosthetic membrane. (**A**) Diffuse distributed lymphocytes (obj. mag. 40×) and (**B**) small perivascular lymphocytic cuff (obj. mag. 20×). Black bar = 50 μm and white bar = 100 μm.

Histological analysis showed granulomatous tissue in a periprosthetic membrane (bone marrow) with macrophages and giant cells. Macrophages contained varying amounts of metal particles in the cytoplasm, which appeared as a grey-blue staining of the cytoplasm or visible black particles (Figure 9). In one case, severe metallosis was found. Besides macrophages and giant cells, lymphocyte infiltration was seen as diffuse infiltration or as small perivascular cuffs (Figure 10).

Periprosthetic tissue histology involves examining the tissue surrounding a prosthetic implant. When metal particles are found in periprosthetic tissue histology, it typically indicates a reaction or response of the tissue to the presence of metal from the prosthetic implant. Common metals in periprosthetic tissue include cobalt, chromium, nickel, titanium, and stainless steel. These particles can vary in size and shape and are seen as black dots in the cytoplasm of macrophages [21]. In preparations stained with hematoxylin and eosin, individual types of metal particles cannot be separated from each other. In this study, it is not possible to separate corundum from other metal particles at the level of histology.

Metal particles can trigger an inflammatory response in the tissue and the presence of immune cells like macrophages and lymphocytes. Lymphocytes are a common finding in histological examinations of tissue surrounding prosthetic implants. They may be present within the granulomatous or connective tissue, around blood vessels, or in areas of inflammation. In addition to the diffusely distributed lymphocytes in the periprosthetic tissue, numerous smaller perivascular clusters of lymphocytes were also seen in this study. The presence of perivascular lymphocytes indicates an immune reaction to the prosthetic implant or its components. The immune response is the body’s way of reacting to foreign material, potentially including metal particles or other constituents of the implant. The number of lymphocytes increases in the periprosthetic tissues with increasing time of implant service [27]. However, the tissue image analysis cannot differentiate finer, potentially metal-induced tissue changes [28].

The interaction of the biomaterial (implant) with its biological environment, the biosystem; the formation of a foreign material-tissue interface; and the long-term success or failure of the integration in the human body are known to be closely correlated with the surface properties of the implant device [12,29].

The composition and the structure of the surface oxide film, the surface contamination, and the surface topography are decisive for the strong osseointegration and prevention of infection and are both required for successful implant survivorship. Gristina et al. described the competition for the free surface between the host’s cells (bone) and bacteria [30]. If the bone cells can reach and occupy the implant surface first, stronger tissue integration will be achieved, and a defensive barrier will also be established against microbial attachment and colonization [16]. Considering the surface science, the Al_2_O_3_ contamination of rough surfaces poses a risk to strong osseointegration and long implant survivorship. The first reports of residual Al_2_O_3_ particles on the surface of a Ti6Al7Nb cementless femoral component of a hip endoprosthesis implant alloy on aseptic loosening and osseointegration appeared around 1990. The authors studied a Ti6Al4V alloy and Ti6Al7Nb rough alloy surface after the grit blasting process with Al_2_O_3_ enrichment, using scanning electron microscopy (SEM), with backscattered BE imaging. They found that the Ti6A7Nb alloy surface was contaminated with up to 25% Al_2_O_3_ debris particles. These references are collected in the Refs. [31,32,33,34,35,36,37,38,39,40,41,42,43].

We were the first to discover that besides implant surfaces, the contamination extends into the subsurface down to 30 μM [16,19,20]. Hard Al_2_O_3_ debris particles (Mosh Scale 9) embed into the soft matrix of Ti6Al7Nb (alpha+ beta) alloy, causing the cracks to spread to the surface. The cracks are potential places for bacteria adhesion and consequently implant infection. Wear debris (metallic, polymeric, and corundum (Al_2_O_3_)) causes inflammation of the soft tissue near the hip endoprosthesis. We found Al_2_O_3_ particles in the soft tissue as determined with histopathology analysis (Figure 9 and Figure 10). The material was not cytotoxic; however, it impacted the growth of the cells.

Several procedures to remove Al_2_O_3_ debris particles from the surface using different acids were developed and introduced into the manufacturing process [31,32]; however, to the best of our knowledge, without any enviable success in cleaning the surface completely.

Our results indicate the importance of the effect of the interaction of particulate matter with the tissues [44,45,46,47,48,49,50,51]; in particular, the interaction of corundum particles with cells. Recommended further research includes the effect of different preparations of corundum yielding particles of different sizes and shapes, investigation of the depth of the penetration of particles into the periprosthetic tissues, the role of corundum nanoparticles on the surfaces, and the effect of corundum nanoparticles on the release of extracellular vesicles from cells. Preparation of the prosthesis surface and possible modifications of this process [52,53] are key in expectations for prosthesis longevity [54].

A clinical implication of this study includes the need for improved prosthesis preparation protocols resulting in less adverse effects such as bacterial-mediated infection and premature wear and failure of prostheses. In practice, this would mean finding the optimal size and shape of corundum particles with respect to the effect that they have on periprosthetic tissues and with respect to the prosthesis surface that interacts with the adjacent cells.

## 4. Conclusions

We have studied the interaction between a hip endoprosthesis composed of Ti6Al7Nb alloy that was subjected to sandblasting with corundum particles, and the adjacent tissue. We found that the Al_2_O_3_ particles penetrated up to 20 μm into the Ti6Al7Nb alloy and we have found metallic particles in the histological slices of the periprosthetic tissue. Al_2_O_3_ did not cause the death of adjacent cells; however, it caused cracks in the matrix of the prosthesis material Ti6Al7Nb alloy and thereby increased the risk for the adhesion of bacteria. This supports our hypothesis that Al_2_O_3_ blasting may be the cause of infection and loosening of hip endoprostheses. As the *in vitro* effects on cells were obtained after 7 days of direct testing, it would be of interest to consider longer times, as well as the effects of Al_2_O_3_ on osseointegration and the use of alternative methods for surface modification of cementless prosthesis stems. Better understanding of the interactions between the blasted surface of the prosthesis and cells is important in tissue engineering to achieve optimal biocompatibility and therefore increase longevity of endoprostheses.

## Figures and Tables

**Table 1:** The results of BMSC growing on Ti6Al7Nb alloys with different surface finishing, observed with confocal 2D/3D laser fluorescence microscopy.

Property	Smooth/Polished	Ground Surface	Rough with Residual Al_2_O_3_ Debris
Number of cells	++	+++	+
Growth orientation	non-oriented	in the grinding direction	direction of the upper surfaces
Cells morphology	it is not pronounced	elongated	porous
Adhesion of cells	good, all surface	good, all surface	partial, locally
Cell condition	growing nicely	growing nicely	cells are not in good condition
Susceptibility to infection	low	low	high

+: few cells, ++: cells in small groups, +++: cells covering the surface.

**Figure A1:**
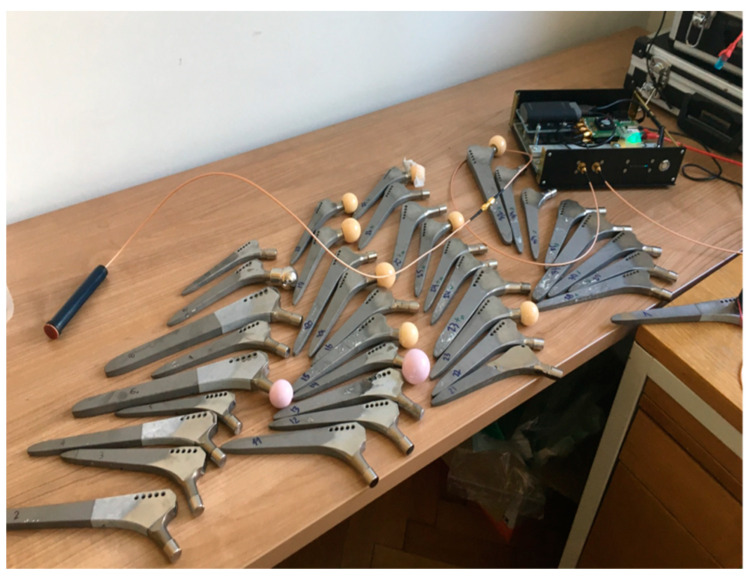
Retrieved prostheses/stems.

## Data Availability

All data are presented in the manuscript.

## Appendix A

Appendix A presents data on retrieved femoral prostheses. More details are presented in [19]. Figure A1 shows the retrieved prostheses/stems and Table A1 presents the list of the retrieved parts and the respective manufacturers. All the retrieved samples, as well as the new ones, were manufactured by ENDOPLUS (London, UK) and Smith&Nephew (London, UK). We also investigated new Zimmer Alloclassic Varial cementless stems.

**Table A1:** Manufacturers and survivorship of retrieved prostheses/parts.

		Manufacturer	Date	Survivorship (Years)	Survivorship (Months)
ASEPTIC LOOSENING (A)					
A1	343	Sminth&Nephew	4 November 2005–8 June 2015	9Y 7M	115
A2	193	ENDOPLUS	19 April 2006–4 November 2013	7 Y 7M	91
A3	221	ENDOPLUS	19 November 2001–28 February 2014	12 Y 3 M	147
A4	379	Sminth&Nephew	12 May 2015–12 June 2016	1 Y	12
A5	41	Sminth&Nephew	15 October 2008–18 August 2011	2 Y 11 M	35
46	120	Sminth&Nephew	7 September 2005–8 January 2013	7 Y 4 M	88
A7	283	ENDOPLUS	20 June 1995–1 June 2015	19 Y 11 M	239
A8	234	Sminth&Nephew	9 April 2014–23 April 2014	14 DAYS	0.5
INFECTION (I)					
I1	184	Sminth&Nephew	25 September 2013–2 December 2013	3 M	3
I2	163	Sminth&Nephew	8 November 2012–12 July 2013	8 M	8
I3	191	Sminth&Nephew	16 October 2013–23 July 2014	9 M	9
I4	171	Sminth&Nephew	19 January 2013–7 August 2013	7 M	7
I5	94	Sminth&Nephew	27 October 2005–16 May 2012	6 Y 5M	77
I6	162	Sminth&Nephew	17 November 2004–16 January 2013	8 Y 2 M	98
I7	40	Sminth&Nephew	8 October 2008–14 September 2011	3 Y	36
I8	129	Sminth&Nephew	26 November 2012–9 February 2013	3 M	3
I9	020	Sminth&Nephew	3 March 2010–29 April 2011	1 Y 1M	13
I10	108	Sminth&Nephew	18 October 2010–2 October 2012	2 Y	24
I11	311	Sminth&Nephew	25 July 2008–11 March 2015	6 Y 6 M	78
I12	205	Sminth&Nephew	6 January 2014–14 January 2014	0.5 M	0.5
LATENT INFECTION (L)					
L1	367	ENDOPLUS	25 September 2002–22 December 2015	13 Y 7 M	163
L2	172	ENDOPLUS	8 October 1998–7 August 2013	14 Y 10 M	178
L3	017	ENDOPLUS	23 March 2005–2 March 2011	6 Y	72
L4	206	Sminth&Nephew	7 November 2008–27 November 2013	5 Y	60
L5	212	Sminth&Nephew	26 May 2011–20 January 2014	2 Y 8 M	32
L6	242	Sminth&Nephew	14 February 2014–15 May 2014	3 M	3
L7	138	Sminth&Nephew	6 November 2007–2 October 2012	5 Y	60

Number of the sample is given in the second column.
